# Metabolomics Analyses Reveal Metabolites Affected by Plant Growth-Promoting Endophytic Bacteria in Roots of the Halophyte *Mesembryanthemum crystallinum*

**DOI:** 10.3390/ijms222111813

**Published:** 2021-10-30

**Authors:** Ryota Kataoka, Mami Akashi, Takeshi Taniguchi, Yoshiyuki Kinose, Ahmet Emre Yaprak, Oguz Can Turgay

**Affiliations:** 1 Department of Environmental Sciences, Faculty of Life & Environmental Sciences, University of Yamanashi, Takeda, Kofu, Yamanashi 400-8510, Japan; nanto.akc@rmail.com (M.A.); ykinose@yamanashi.ac.jp (Y.K.); 2 Aridland Research Center, Tottori University, Hamasaka, Tottori 680-8550, Japan; takeshi@tottori-u.ac.jp; 3 Department of Biology, Faculty of Science, Ankara University, Ankara 06560, Turkey; emre.yaprak@science.ankara.edu.tr; 4 Department of Soil Science and Plant Nutrition, Faculty of Agriculture, Ankara University, Ankara 06560, Turkey; o.can.turgay@agri.ankara.edu.tr

**Keywords:** *Mesembryanthemum crystallinum*, endophytic bacteria, plant growth-promoting bacteria, metabolome, *Microbacterium* spp., *Streptomyces* spp., salinity

## Abstract

*Mesembryanthemum crystallinum* L. (common ice plant) is an edible halophyte. However, if ice plants are used to phytoremediate salinity soil, there are problems of slow initial growth, and a long period before active NaCl uptake occurs under higher salinity conditions. Application of endophytic bacteria may improve the problem, but there remain gaps in our understanding of how endophytic bacteria affect the growth and the biochemical and physiological characteristics of ice plants. The aims of this study were to identify growth-promoting endophytic bacteria from the roots of ice plants and to document the metabolomic response of ice plants after application of selected endophytic bacteria. Two plant growth-promoting endophytic bacteria were selected on the basis of their ability to promote ice plant growth. The two strains putatively identified as *Microbacterium* spp. and *Streptomyces* spp. significantly promoted ice plant growth, at 2-times and 2.5-times, respectively, compared with the control and also affected the metabolome of ice plants. The strain of *Microbacterium* spp. resulted in increased contents of metabolites related to the tricarboxylic acid cycle and photosynthesis. The effects of salt stress were alleviated in ice plants inoculated with the endobacterial strains, compared with uninoculated plants. A deeper understanding of the complex interplay among plant metabolites will be useful for developing microbe-assisted soil phytoremediation strategies, using *Mesembryanthemum* species.

## 1. Introduction

Salinity, which is a major environmental issue in arid and semi-arid regions, imposes stress upon vegetation and exacerbates the effects of other stresses, including water scarcity, nutrient deficiencies, and soil alkalinity [1]. It is estimated that of the 230 million ha of irrigated land, 45 million ha (19.5%) is salt affected [2]. In addition, it is thought that 20% of the global irrigated area is affected by salinization caused by irrigation. In some countries, such as Egypt, Iran and Argentina, salinized soils constitute more than 30% of the irrigated land [3,4]. The world’s population is expected to reach 9.7 billion by 2050 [5], and there are expected to be serious issues with food supply. Therefore, to ensure stable food supply, the development of efficient technologies to remediate and regenerate dry and salinized agricultural land is an urgent challenge [6]. Salt-removal technologies include physical–chemical methods, such as gypsum-based sodic soil reclamation, and subsurface drainage of water-logged saline soils to remove salts accumulated at the surface [7]. However, these methods are costly and may have other negative effects on the environment [8]. Under such conditions, phytoremediation can be considered a cost-effective and environmentally friendly technology for soils suffering from salinity and alkalinity problems [9]. Wu et al. [10] reported that hydrocarbon-degrading and plant growth-promoting bacterial endophytes were effective to facilitate the phytoremediation of petroleum-contaminated soils with high salinity. Moreover, many other halophytes, such as *Sedum aizoon* L. [11], *Suaeda salsa* [12], *Suaeda maritima* and *Sesuvium portulacastrum* [13]), and *Salicornia ramosissima* [14], are reported to be favorable plants that could be used for the phytoremediation of saline- and heavy metals–contaminated soil.

*Mesembryanthemum crystallinum* L. (common ice plant) is a salt-tolerant and edible plant that can grow under high-salt conditions, such as in soils irrigated with seawater, and under harsh dry conditions. These plants accumulate salt in giant epidermal bladder cells [15]. Because the ice plant is an edible halophyte, it can be grown agriculturally and can also be an option for the phytoremediation of saline soils. Therefore, agricultural production can be maintained if it is used to remediate soil. However, ice plants are only moderately salt tolerant (withstanding up to 150 mM NaCl in soil) at the seedling stage, whereas mature plants can tolerate high concentrations of salt (withstanding > 450 mM NaCl in soil) [16]. If ice plants are taken into consideration as a phytoremediation agent for saline soils, it would be favorable to promote their germination and early growth so that plants attain maturity more effectively and rapidly. In the present study, we therefore searched for plant growth-promoting endophytic bacteria (PGPE) that may promote (or stimulate) the initial growth of ice plants. For this strategy to be effective, it is important that PGPE are maintained at sufficient population sizes in the rhizosphere and nearby soils. The soil in which plants grow is already inhabited by microbes, so even if selected beneficial bacteria are introduced to the soil, it may be difficult for the introduced microbes to establish because of competition from indigenous microbes [17]. Therefore, we speculated that the growth-promoting effect could be maintained stably by using endophytic microbes that settle stably regardless of environmental factors. To the best of our knowledge, only two previous studies have isolated endophytic bacteria from ice plants. The aims of those studies were to investigate the effects of the isolated microbes on plant growth and to isolate key functional genes involved in salt tolerance [18,19]. Thus, gaps remain in our understanding of the extent of endophytic bacteria in ice plants, their growth-promoting activities, and their effects on the biochemical and physiological responses of the plants. In the present study, endophytic bacterial strains producing an important plant hormone, indole-3-acetic acid (IAA), were isolated, and their ability to promote initial growth of ice plant seedlings was evaluated. IAA is the most common bioactive endogenous auxin and participates in diverse aspects of plant growth and development [20]. IAA is produced in the seed and young leaves, for example [21]. It has a significant impact on crops by increasing nutrient uptake through the development of longer roots and increasing the number of lateral root hairs [22]. In addition, it is known that the metabolic changes of plants occur in response to salt stress, mainly including plant–pathogen interaction, amino acid metabolism of the beta alanine, arginine, proline and glycine metabolism, carbon metabolism of glycolysis, gluconeogenesis, galactose, starch and sucrose metabolism, plant hormone signal transduction and spliceosome [23,24]. Therefore, a metabolomics analysis was conducted to solve one of the gaps. Metabolomics can assist in attaining a thorough understanding of the complex plant metabolic networks, and their responses to environmental and genetic change [25]. Thus, the aims of this study were to identify PGPE from ice plants and to document the metabolomic response of salt-stressed and control ice plants to these endophytic bacteria.

## 2. Results

### 2.1. Plant Growth and Plant Microbiome under Saline Conditions

The ice plants could grow in soil containing 50 mM and 200 mM NaCl. However, when the NaCl concentration reached 350 mM, ice plant growth was inhibited in all three soils (Figure S1). The fresh weight of the above-ground parts of the ice plants was higher in the 50 mM and 200 mM NaCl treatments than in the other treatments, and was highest in soil from Atagoyama, followed by soil from the university farmland, and lowest in the soil from Kofu. A significant difference was observed in the fresh weight of the above-ground parts among the three types of soil containing 50 mM NaCl. This may be due to not only the soil microbial characteristics, but also the soil physicochemical features.

**Figure 1:**
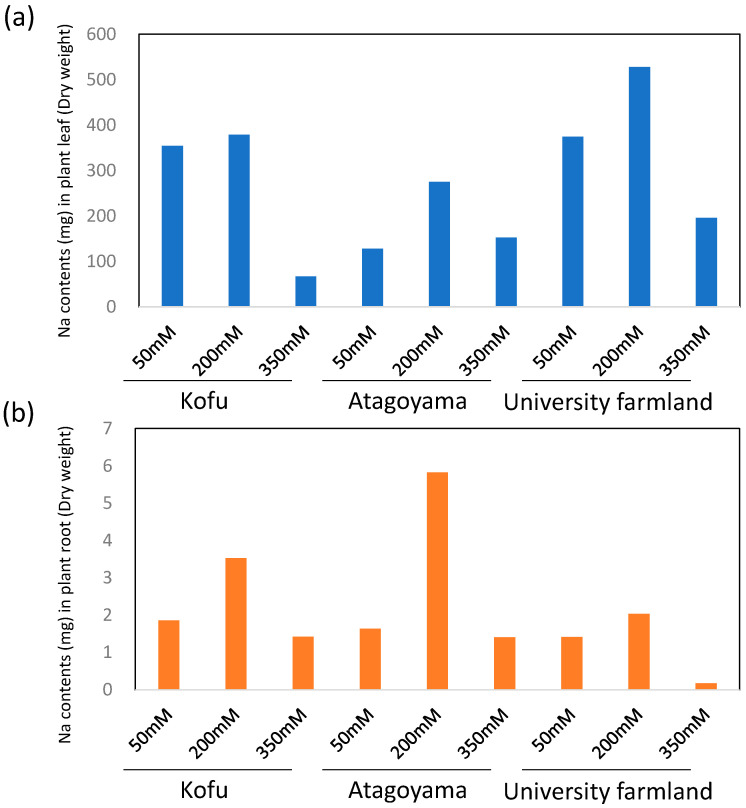
Amount of sodium taken up by ice plants under different salinity conditions. (**a**) Leaves, (**b**) roots.

The amount of Na taken up per plant was highest in the 200 mM NaCl treatment and lowest in the soils containing 350 mM NaCl (Figure 1).

**Figure 2:**
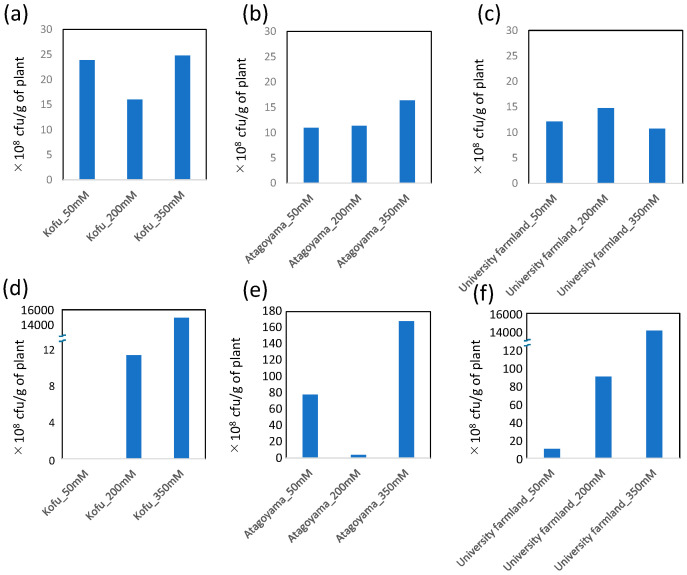
Microbes obtained from rhizosphere soil (**a**–**c**). Endophytic bacterial colony forming units of microbes isolated from ice plants growing in different soils (**d**–**f**). Values are means (*n* = 2).

**Figure 3:**
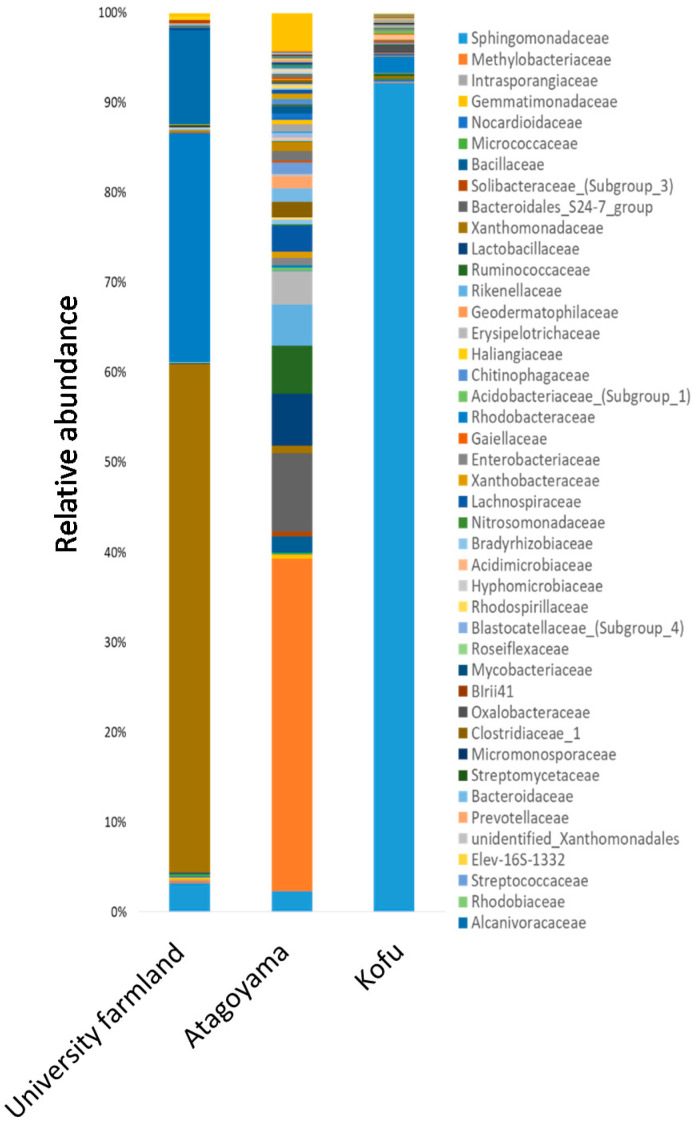
Relative abundance (based on total sequence reads) of endophytic bacterial families in microbiomes of ice plants grown in three different soils in a pot experiment.

In all soils, the number of endophytic bacteria increased in parallel with the salt concentration applied. However, the number of rhizosphere bacteria was similar between treatments, regardless of salinity (Figure 2). The endophytic bacterial composition in the three different soils containing 350 mM NaCl was as follows: university farmland soil, 56.5% Xanthomonadaceae, 25.55% Rhodobacteraceae, and 10.41% Alcanivoracaceae; Atagoyama soil, 36.92% Methylobacteriaceae, 8.67% Bacteroidales_S24-7_group, 5.77% Lactobacillaceae, and 5.34% Ruminococcaceae; Kofu soil, 92.23% Sphingomonadaceae (Figure 3).

### 2.2. Plant Growth-Promoting Endophytic Bacteria

A total of 64 salt-tolerant PGPE strains (14 from Kofu soil, 25 from Atagoyama soil, and 25 from university farmland soil) were isolated from the soils cultured on medium containing 513 mM NaCl. Ten strains with high IAA-producing ability were selected by Salkowski’s test. In addition to these 10 strains, 1 strain isolated from an ice plant growing in sandy soil collected from Tottori Prefecture (35°53′71″ N, 134°21′67″ E) was also used for further experiments. Characteristically, the strains with high IAA production isolated from the Kofu, Atagoyama, and university farmland soils tended to be abundant around the roots of ice plants growing in soil containing 350 mM NaCl. To evaluate the effects of these strains on the initial growth of the ice plant, two strains (strains 2 and 4) were selected for further analysis. Only strain 4, which was isolated from Atagoyama soil with 350 mM NaCl, showed IAA production. None of the other plant growth-promoting traits were detected in either strain.

The 16S rRNA sequences of strains 2 (accession no. LC640671) and 4 (accession no. LC640670) were compared with other bacterial nucleotide sequences in GenBank. Strains 2 and 4 exhibited high sequence similarity to *Streptomyces* spp. and *Microbacterium* spp., respectively. The strain with the highest sequence similarities to strain 4 was *Microbacterium xylanilyticum* strain C10 (MF872640), and that with the highest similarity to strain 2 was *Streptomyces thermocarboxydus* strain EGI124 (MN704433).

**Figure 4:**
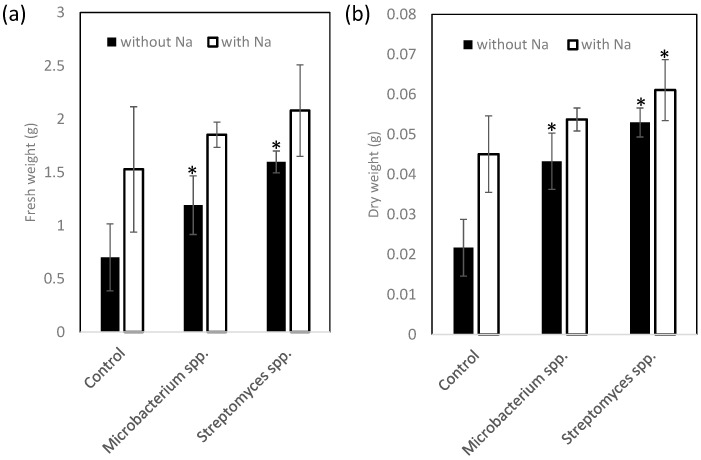
Effect of *Streptomyces* spp. strains 2 and *Microbacterium* spp. strain 4 on growth of ice plants in the control (no NaCl) and under 200 mM NaCl. (**a**) Fresh weight and (**b**) dry weight of whole plant. Closed bar indicates 0 mM NaCl, open bar indicates 200 mM NaCl. Values are mean ± standard deviation (*n* = 3–6). * Indicates a significant difference between a treatment and control (Dunnett’s test, *p* < 0.05).

A vial experiment was conducted to evaluate the effects of the selected PGPE strains on the growth of the ice plants. Plant fresh and dry weights were significantly affected by the strains (one-way ANOVA, *p* < 0.05). Plants inoculated with strains 2 and 4 showed significantly better growth than did the control (Dunnett’s test, *p* < 0.05). Compared with the control, plants inoculated with strain 2 showed significantly better growth under saline conditions, whereas those inoculated with strain 4 did not (Figure 4, Figure S2).

### 2.3. Metabolome Analysis of PGPE Inoculated Ice Plant

**Figure 5:**
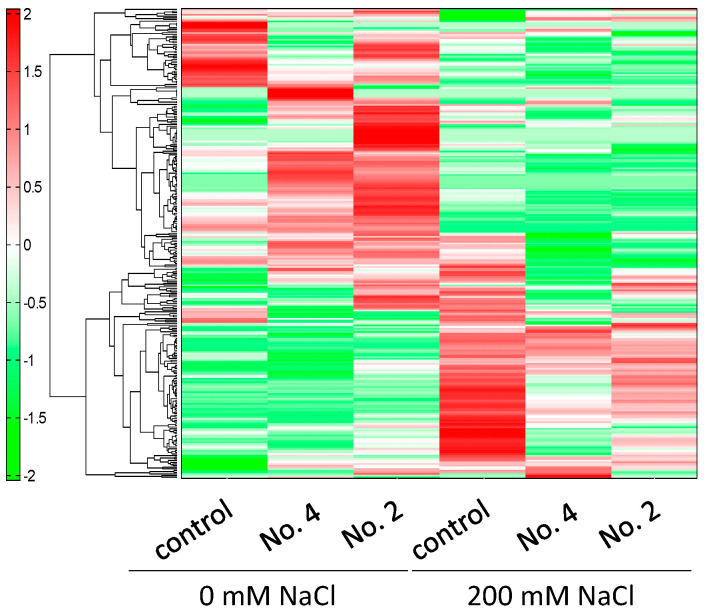
Heatmap of all metabolites based on relative peak area in mock-inoculated ice plants and those inoculated with strain 4 and strain 2. Horizontal axis and vertical axis show sample names and peaks, respectively. Distances between peaks are displayed in the tree diagram. Green (low) to red (high) represent the increase in relative area of each peak among the three treatments. Data in this figure correspond to Table S1.

**Figure 6:**
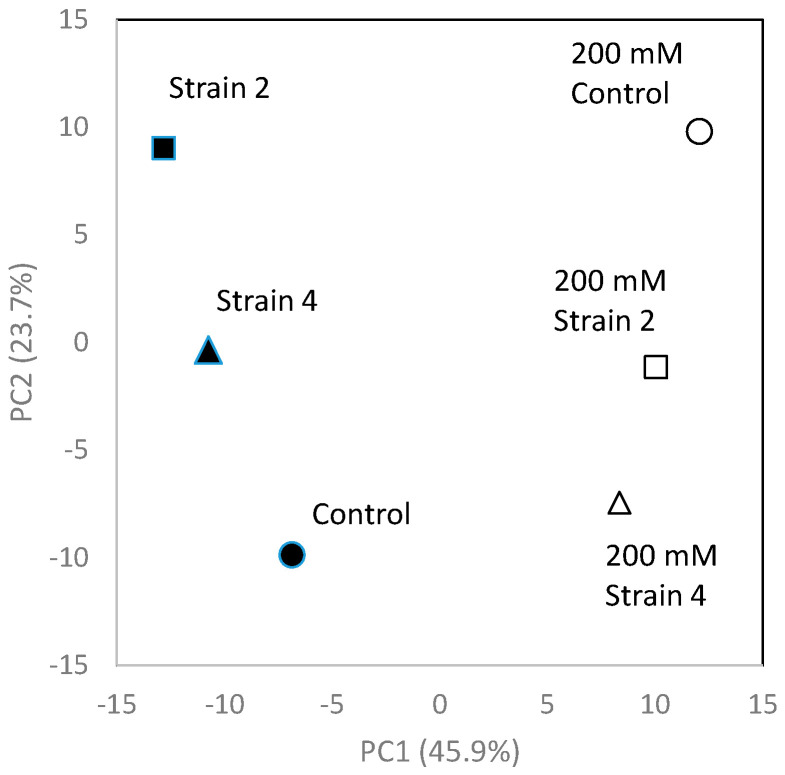
Scaling principal component analysis (PCA) based on metabolites detected in ice plants grown with/without NaCl and inoculation with strain 2 or strain 4 or mock inoculation. Open symbol and closed symbol indicate 200 mM and 0 mM NaCl, respectively. All samples and annotated metabolites were included in the PCA.

**Figure 7:**
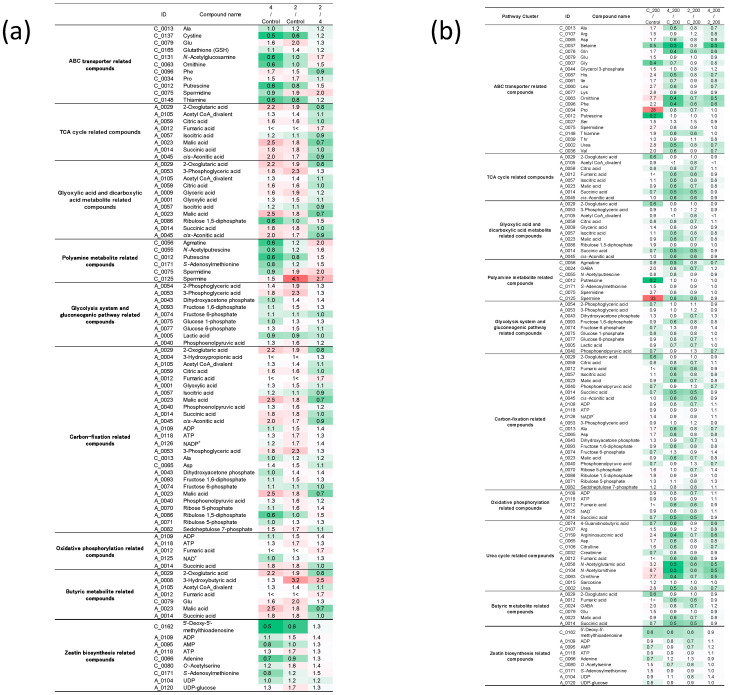
Heatmap of major metabolite groups based on comparative ratio in plants inoculated with strain 4 or strain 2 compared with mock-inoculated plants (**a**); and in plants inoculated with strain 4 or strain 2 compared with mock-inoculated plants under saline conditions (**b**). Green (low) to red (high) represents the increase in relative area of each peak among the three treatments.

Next, CE-TOFMS analyses of common ice plant were carried out in cation and anion modes. In this study, analysis of substances registered in the HMT Metabolite Library and the Known–Unknown Peak Library was conducted. We detected 281 metabolites (166 cationic and 115 anionic). Selected data are shown in Figure 5, Figure 6 and Figure 7. The relative peak area of all observed metabolites differed among the three treatments. Of the 281 metabolites, 49.5% and 68.3% were more abundant in ice plants grown with strain 4 and strain 2, respectively, than in control plants. Of all the metabolites, 27.8% were more abundant in plants grown with one strain but less abundant in plants grown with the other strain, compared with the control; 16.4% were less abundant in plants grown with PGPE strains than in the control; 13.2% were not detected in the control, but only in plants grown with one of the PGPE; and 2.1% were detected in the control, but not in the plants grown with PGPE. We also detected differences in metabolite profiles between control plants and those under salinity stress. Of the 281 metabolites, 54.4% were more abundant in salt-stressed plants than in control plants; 6.8% were more abundant in plants grown with one PGPE strain and less abundant in plants grown with the other PGPE strain, compared with uninoculated plants under salinity stress; and 47% were less abundant in plants grown with PGPE than in mock-inoculated plants under salinity stress. A principal component analysis (PCA) score plot constructed from CE-TOFMS data separated the treatments into two distinct groups with respect to salinity (with/without NaCl) and three distinct groups with respect to PGPE (stain 2, strain 4, and no PGPE) on PC 1 (45.9%) and PC 2 (23.7%) (Figure 6). The factor loadings within each principal component of the PCA suggested the explanatory factors: the values of N1-acetylspermine, guanosine, lysine, and proline increased under salt treatment, while the values of inosine, GDP-mannose, GDP-glucose, O-acetylhomoserine-1, and ethanolamine phosphate decreased under salt treatment. Glycerol was extracted as an explanatory factor for PC2. Its value was lower in plants grown with strains 2 and 4 than in mock-inoculated plants under non-saline conditions, but was higher in plants grown with strains 2 or 4 than in mock-inoculated plants under saline conditions. Compared with mock-inoculated plants, ice plants grown with strains 2 and 4 showed increased contents of 2-oxoglutaric acid, acetyl CoA_divalent, citric acid, fumaric acid, isocitric acid, malic acid, succinic acid, and cis-aconitic acid, which are involved in the TCA cycle. In particular, fumaric acid was more abundant in plants grown with strain 2 than in those grown with strain 4. The abundance of ADP, ATP, and NADP^+^, which are important for photosynthesis and oxidative phosphorylation, was 1.5 times higher in plants grown with strain 2 than in mock-inoculated plants. Plants grown with strain 2 and those grown with strain 4 showed increased contents of 3-phosphoglyceric acid, malic acid, and sedoheptulose 7-phosphate, which are important for carbon fixation. Plants grown with strain 2 also showed increased contents of phosphoenolpyruvic acid and ribose 5-phosphate. The abundance of UDP-glucose, which is required for synthesizing sucrose as the energy source for plants, was 1.7 times higher in plants grown with strain 2 than in mock-inoculated plants. Plants grown with strain 2 also showed increased contents of 2-oxoglutaric acid, 3-phosphoglyceric acid, acetyl CoA_divalent, citric acid, glyceric acid, glycolic acid, glyoxylic acid, isocitric acid, malic acid, succinic acid, and cis-aconitic acid. These results suggested that strain 2 activated the glyoxylic acid pathway in ice plants, possibly to derive glucose as an energy source. Compared with plants under control conditions, those grown under salinity stress (200 mM NaCl) showed increased contents of ABC transporter–related compounds, proline, and spermine.

## 3. Discussion

Plant microbiome communities consist of large numbers of bacteria, including ectophytes and endophytes. These microbes provide critical and sustainable benefits to improve soil health and nutrient utilization and increase plant growth and development [26]. To isolate endophytic bacteria, ice plants were grown in three types of soils under different salt treatments. The first step to screen for PGPE was to analyze IAA production, but the results showed that IAA concentration may not always be directly linked to the plant growth-promoting effect. Focusing only on growth-promoting traits may not be useful for the screening of isolates. However, to select strains with a plant growth-promoting effect from many isolates, it is one way to detect specific functions. It is important to evaluate the effects of each strain on plant growth to determine its plant growth-promoting ability.

The sequencing results revealed that different soils significantly affected the endophytic bacterial community within ice plants grown under saline conditions (Figure 3). The endophytic bacterial community is a subset of the rhizosphere and/or root-associated bacterial population [27,28]. Therefore, differences in endophytic bacterial communities among the three sites may be due to differences in the bacterial communities of the soils. Interestingly, salt concentrations of the soils in the present research were not high, as shown by EC (0.07–0.31 mS cm^−1^), but the number of endophytic bacteria isolated from ice plants grown in all soil types increased as the salt concentration increased (Figure 2). These findings suggested that some soil microorganisms in low-salinity soils potentially possess high salinity tolerance and invade the roots of ice plants under saline conditions. The enrichment of bacteria by ice plants under saline conditions may be one mechanism by which ice plants adapt to stressful conditions, as suggested by Liu et al. [29].

Many PGPE have multiple functions, such as IAA production and phosphate-solubilization activity [30]. However, of the two strains selected in our study, only strain 4 showed IAA production. Strain 2 promoted initial plant growth but did produce IAA, nor did it give a positive result in any of the other tests. This raises the possibility that its growth-promoting mechanism may differ from those previously reported.

**Figure 8:**
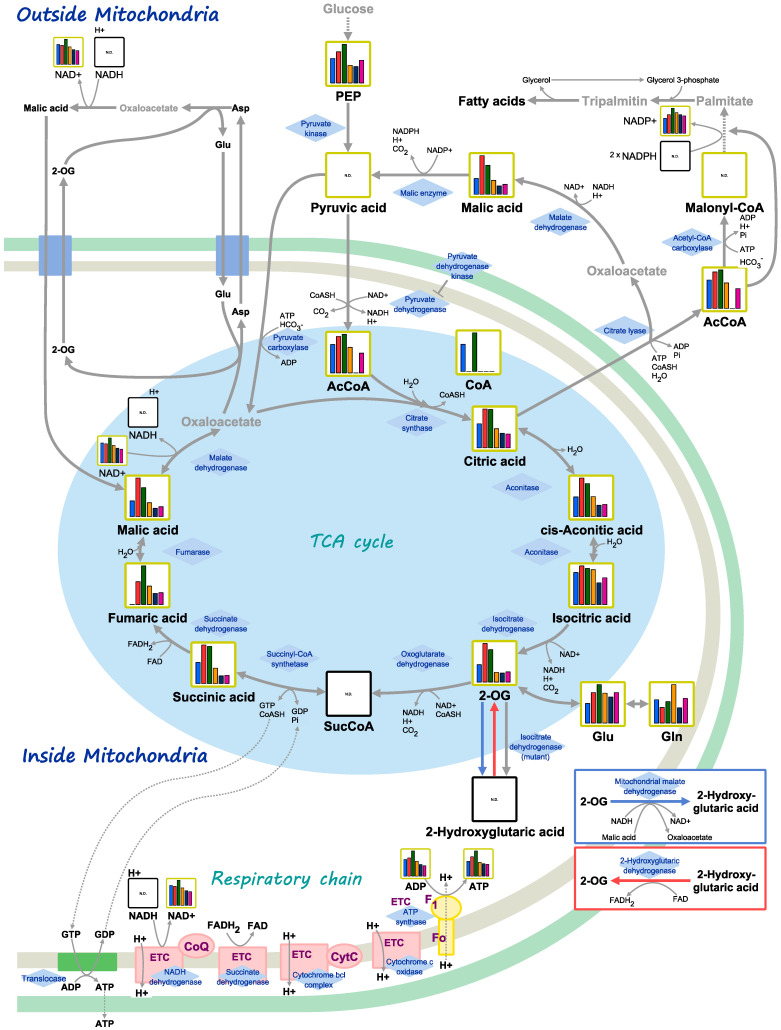
Location of substances detected in this study in the metabolic pathway. Substances were detected by comparison with metabolites registered in the HMT metabolite library. Graph shows the relative area values in mock-inoculated control (blue), plants inoculated with strain 4 (red), plants inoculated with strain 2 (green), mock-inoculated control plants under saline conditions (orange), plants inoculated with strain 4 under saline conditions (dark blue), and plants inoculated with strain 2 under saline conditions (purple). N.D: not detected.

The CE-TOFMS metabolic profiling analyses highlighted differences in metabolic profiles between PGPE-inoculated and mock-inoculated plants, and between salt-stressed and control plants. We detected differences in metabolic profiles related to bacterial strains and to salinity. Both PGPE strains affected plant metabolism (Figure 5), consistent with the large metabolic changes caused by endophytic bacteria detected in our previous study [31]. Thus, the results of the present study confirm that endophytic bacteria strongly affect plant metabolism. In plants, interactions with endophytic bacteria lead to changes in primary metabolic pathways, such as energy production and the biosynthesis of precursors for secondary metabolism [32,33]. In the present study, ice plants grown with strains 2 and 4 showed increased abundance of 2-oxoglutaric acid, acetyl CoA_divalent, citric acid, fumaric acid, isocitric acid, malic acid, succinic acid, and *cis*-aconitic acid, which are involved in the TCA cycle (Figure 8). The main function of the TCA cycle is to generate ATP, which provides energy for cellular processes. Increases in TCA flux require increased amounts of substrates for the enzymatic reactions in this cycle [34]. Our findings suggest that PGPE, especially strain 2, may activate the metabolism in ice plants. Moreover, the TCA-related compounds showing increased abundance in the PGPE-inoculated plants play important roles in photosynthesis, photorespiration, nitrogen metabolism, reductant transport, and maintenance of the photosynthetic redox balance [35]. A previous study found that PGPE could enhance plant photosynthetic activity [36]. Consistent with this, ice plants inoculated with strain 2 showed increased contents of ADP, ATP, NADP^+^, 3-phosphoglyceric acid, malic acid, phosphoenolpyruvic acid, ribose 5-phosphate, and sedoheptulose 7-phosphate, which are involved in photosynthesis. This effect was stronger in plants inoculated with strain 2 than in those inoculated with strain 4, and may be related to the up-regulation of the TCA cycle in plants inoculated with strain 2. Malic acid/malate was reported to be involved in plant-endophytic bacteria communication in roots [37]. Interestingly, ADP, AMP, ATP, adenine, *O*-acetylserine, *S*-adenosylmethionine, UDP, and UDP-glucose, which are involved in the biosynthesis of the plant hormone zeatin, were also more abundant in the plants inoculated with strain 2 than in uninoculated plants. We did not detect increased contents of zeatin, but previous studies have detected increased zeatin production in plants infected with endophytes [38,39]. In the current study, metabolomics analyses were conducted on 11-day-old seedlings. In future research, it will be important to evaluate the longer-term effects of strain 2 on the metabolome of ice plants.

Plants alleviate salt stress effects through various mechanisms, such as ion transport and uptake, biosynthesis of compatible solutes, synthesis of polyamines, and adaptive regulation by stress-related hormones [40,41]. In the present study, saline conditions affected the metabolome of ice plant seedlings. Salt stress resulted in increased contents of ABC transporter–related compounds. The ABC transporters are involved in several aspects of plant growth and adaptation, including the mobilization of plant hormones [42] and the maintenance of ionic homeostasis [43]. Different ABC transporter genes (*ABCB21*, *ABCG36*, and *ABC2*) were found to be up-regulated under salinity stress [44]. However, compared with mock-inoculated plants under salt stress, salt-stressed plants grown with strain 2 or 4 showed decreased contents of ABC transporter-related compounds, suggesting that both strains may have relieved the negative effects of salt stress.

We detected a 28-fold increase in proline content in salt-stressed plants, indicating that proline accumulated as a compatible solute in response to salt stress. Proline reacts with and detoxifies reactive oxygen species; thus, its accumulation may be an important factor to maintain high photosynthetic rates under stress conditions [45]. In addition, the content of the polyamine spermine was 33-fold higher in salt-stressed plants than in the control plants. Spermine and spermidine are polyamines that are closely associated with the intracellular ion balance and the metabolism of the compatible solute, proline. Their intracellular concentrations fluctuate significantly during salt stress. Moreover, salt stress also results in oxidative stress, which is intimately associated with mitochondrial metabolism and necessary for the assimilation of nitrate, which is required for glutamate production [46]. In another study, increased activity of the TCA cycle was not detected under osmotic stress [47]. However, in this study, salt-stressed plants showed reduced contents of compounds involved in the TCA cycle, except for glutamic acid, which was 1.5-times higher in salt-stressed plants than in control plants.

We detected increased contents of cyclic 3′,5′-adenosine monophosphate (cAMP) in salt-stressed plants, compared with those of control plants. Cyclic AMP is a second messenger that is present in almost all living organisms. It plays a pivotal role in cell signaling and modulates a variety of cellular responses [48]. Maathuis and Sanders [49] found that the addition of membrane-permeable cyclic nucleotides improved the salt tolerance of plants, as evaluated using plant growth assays. Our findings suggest that cAMP also plays an important role in the response of ice plants to salinity stress.

## 4. Materials and Methods

### 4.1. Ice Plant Growth and Sodium-Uptake Ability under Saline Conditions

Common ice plants were grown to evaluate their growth and to isolate endophytic bacteria under different salinities. We isolated endophytic bacteria from three different soil samples collected in Yamanashi prefecture, Japan in February 2019 (Atagoyama, temperate deciduous forest; 35°67′20″ N, 138°58′16″ E, Kofu, cultivated field; 35°67′86″ N, 138°57′11″ E, university farmland soil, cultivated soil; 35°60′34″ N, 138°57′88″ E). These three soil samples were collected from different environmental conditions and represented different soil types (Cambisols, Andisols, and Fluvents, respectively). Atagoyama and Kofu are 1.56 km apart in a straight line, and Kofu and the university farm are 8.78 km apart in a straight line. The above sea level of Atagoyama is slightly higher (approximately 300 m) than other two sampling sites (250 m). The chemical properties of the sampled soils are summarized in Table 1.

We collected approximately 5–10 kg soil (5–20 cm soil depth) from each of the three sites. Soil samples were collected from sampling points free of plants, although weeds were growing near the sampling point at Atagoyama. After sieving the collected soils (<φ2 mm), 350 g soil was added to each Neubauer pot (100 cm^2^; Fujiwara Seisakusho, Ltd. Tokyo, Japan). The soil water content was adjusted to 60% of the maximum water-holding capacity. Ice plant seeds were sown in the pots, and seedlings were grown for 3 weeks. Then, at 1 week intervals, saline solution was added to achieve final Na concentrations of 50 mM, 200 mM, and 350 mM by NaCl solution with liquid fertilizer (Hyponex, HYPONeX Japan Corp., Osaka, Japan). The plants were then grown for a further 6 weeks. After 10 weeks of growth in total, the fresh weights and dry weights of the above-ground parts and roots of the ice plants were determined. To determine sodium (Na) uptake, the leaves and roots of the harvested ice plants were separately dried and crushed. Then, a 0.1 g sample (leaf or root) was placed in a 15 mL tube, 750 µL nitric acid was added in accordance with the modified method of Yamaki [50], and the mixture was incubated for 30 min. MilliQ water was added to complete the volume to 15 mL, and then the mixture was shaken for 30 min. After filtration, the solution was diluted 50-fold, and the amount of Na absorbed was measured, using a microwave plasma atomic emission spectrophotometer (Agilent Technology 4100 MP-AES, Agilent Technologies Japan, Ltd., Tokyo, Japan).

### 4.2. Microbiome of Ice Plants

Total DNA was extracted from 0.1 g of plant root tissues, which were prepared as a pooled sample after collection from three roots within the same treatment and surface-sterilized with 70% ethanol and 1% NaOCl to examine endophytic bacteria-related DNA [51], using the FastDNA™ Spin Kit for Soil (MP Biomedicals, Tokyo, Japan). The DNA concentration was checked, using a BioSpec-nano spectrophotometer (Shimadzu Corporation, Kyoto, Japan) and then the DNA was diluted to a concentration of 1 ng μL^−1^ using sterile water. For barcoding, the V4 region of the 16S rRNA gene was amplified using specific primers: 515F (5ʹ-GTGCCAGCMGCCGCGGTAA-3ʹ) and 806R (5ʹ-GGACTACHVGGGTWTCTAAT-3ʹ). All PCR reactions were carried out with the Phusion^®^ High-Fidelity PCR Master Mix (New England Biolabs Japan Inc., Tokyo, Japan). For quantification and quality control, the PCR products were mixed with an equal volume of 1× loading buffer (containing SYBR green) and then separated by electrophoresis on a 2% *w*/*v* agarose gel. Samples producing clear bands with an approximate size of 400–450 bp were chosen for further experiments. The PCR products were mixed at equal density ratios. Thereafter, the mixed PCR products were purified using a Qiagen Gel Extraction Kit (Qiagen, Hilden, Germany). The libraries and the 250 bp paired-end reads, which were generated with the NEBNext^®^ Ultra™ DNA Library Prep Kit for Illumina and quantified by Qubit fluorometry and quantitative PCR, were sequenced on the Illumina HiSeq 2500 platform (Illumina, San Diego, CA, USA). To verify the reliability of the data, quality control was performed at each step of the procedure. Paired-end reads were assigned to samples based on their unique barcode and truncated by removing the barcode and primer sequences. Paired-end reads were merged using FLASH (V1.2.7) [52]. Quality filtering of the raw tags was performed under specific filtering conditions to obtain high-quality clean tags [53], according to the quality control process in QIIME (V1.7.0) [54]. The tags were compared with the reference database (Gold database) using the UCHIME algorithm [55] to detect chimera sequences (http://www.drive5.com/usearch/manual/chimera_formation.html). The chimera sequences were removed [56] to obtain the final effective tags. Sequence analysis was performed using Uparse software (V7.0.1001), using all the effective tags [57]. Sequences with ≥97% similarity were assigned to the same operational taxonomic unit (OTU) [58]. A representative sequence for each OTU was screened for further annotation. For each representative sequence, Mothur software was used against the SILVA SSU rRNA database [59] for species annotation at each taxonomic rank (threshold: 0.8~1) [60]. To explore the phylogenetic relationships among all OTU representative sequences, MUSCLE software (V3.8.31) was used for rapid comparison of multiple sequences [61]. The OTUs abundance information was normalized, using a standard of sequence number corresponding to the sample with the fewest sequences. Subsequent analyses were performed based on these output-normalized data. The abundance data of sequences matching “Chloroplast” and “Mitochondria” were archived, and these sequences were removed from the datasets. The refraction curve was created by randomly selecting a specific amount of sequence data from a sample and counting the number of species they represented. The reads have been submitted to the DDBJ Sequence Read Archive (https://www.ddbj.nig.ac.jp/dra/index-e.html) under Bioproject and are available under the accession number DRA012381.

#### 4.2.1. Isolation of Endophytic Bacteria

Endophytic bacteria were isolated from harvested ice plants in accordance with the slightly modified method of Navarro-Torre et al. [62] and Egamberdieva et al. [63]. Half of the first leaf and 0.01 g of the root were sterilized by immersion in 1% sodium hypochlorite for 5 min and then 70% ethanol for 1 min, following by several washes with sterilized water. After sterilization, each sample was homogenized with a mortar and pestle. Then, 50 μL of the suspension was spread onto Reasoner’s 2A agar (R2A) (Eiken Chemical Co., Ltd., Tochigi, Japan) medium (pH 7.0) containing 513 mM NaCl. The plates were kept in an incubator at 25 °C for 3 days. Colonies with different morphologies were visually selected for further analysis. To confirm that the surface sterilization process was successful, the last rinse solution was inoculated onto a R2A medium and cultured at 25 °C for 72 h. The absence of bacterial growth confirmed that the surface sterilization was successful [51]. Therefore, re-isolation was not conducted in this study.

#### 4.2.2. First Screening Step for Plant Growth-Promoting Endophytic Bacteria

The first screening step was to evaluate the ability of isolated bacteria to produce the plant hormone IAA. The production of IAA was evaluated, using Salkowski’s reagent [64,65]. The strains were grown for 96 h at 25 °C in IAA production medium (30 g glucose, 2 g beef extract, 3 g CaCO_3_, pH 7, and 1 L sterile H_2_O) with and without 1 mM tryptophan. The cultures were harvested by centrifugation at 10,000× *g* for 10 min. A 300 μL aliquot of the supernatant was mixed with 1.2 mL Salkowski’s reagent. The absorbance of the mixture at 535 nm was measured with a spectrophotometer.

#### 4.2.3. Second Screening Step for Plant Growth-Promoting Endophytic Bacteria

All the strains showing IAA production and a strain collected from Tottori Prefecture in 2019 were screened to determine their plant growth-promoting ability, using the modified method of Liu et al. [66]. Each selected endophytic bacterium was inoculated using a disposable loop into 2 mL potato dextrose broth (PDB) medium (adjusted to pH 6.5 with 0.1 M NaOH) (Difco, Japan BD, Tokyo, Japan) with or without 1 mM of tryptophan in a 15 mL centrifugation tube. The culture was incubated in a shaking incubator at 25 °C for 24 h. Seven ice plants seeds (surface-sterilized by immersion in 1% sodium hypochlorite for 30 s, then in 70% ethanol for 30 s, and then rinsed twice) were placed in the culture medium and incubated at 25 °C for 48 h. The seeds and medium were then spread on an autoclaved filter paper in a Petri dish and grown for 1 week. The fresh weight and root length of the ice plants after 1 week were measured to evaluate the growth-promoting effect of each bacterial strain.

#### 4.2.4. PGPE Characterization and Phylogenetic Analysis of Selected Strains

Phosphate solubilization, siderophore production, and the transcript levels of *nif*H and the gene encoding 1-aminocyclopropane-1-carboxylate-deaminase (ACCd) were studied in the selected strains, using standard procedures.

To evaluate phosphate solubilization, each strain was cultured on Pikovskaya’s medium [67], which contained tri-calcium phosphate, for 7 days at 25 °C. A clear zone was indicative of phosphate solubilization, and the results were scored as follows: −, no clear zone; +, detectable but weak clear zone; ++, obvious clear zone.

For the siderophore production assay, each strain was cultured on chrome azurol S (CAS) medium [68,69] and then overlaid with the color indicator medium (6.04 mg CAS, 7.3 mg hexadecyltrimetyl ammonium bromide, 3.04 g piperazine-1,4-bis(2-ethanesulfonic acid), and 1 mL of 1-mM FeCl_3_·6H_2_O). The color change of CAS from blue to light orange or yellow was indicative of siderophore production, and the results were scored as follows: −, no color change; +, color change; ++, color change of entire medium.

The DNA was extracted from selected strains, using a ZR Fungal/Bacterial DNA MiniPrep Kit™ (Zymo Research, Irvine, CA, USA) and then PCR amplification of the *nif*H and ACCd genes was carried out using a T100™ Thermal Cycler (Bio-rad, Hercules, CA, USA). The thermal cycling conditions were as follows: one cycle at 94 °C for 4 min; 30 cycles at 94 °C for 60 s, 54 °C for 60 s, and 72 °C for 2 min; final extension at 72 °C for 7 min for ACCd; one cycle at 94 °C for 5 min; 30 cycles at 94 °C for 60 s, 55 °C for 60 s, and 72 °C for 2 min; and final extension at 72 °C for 5 min for *nif*H. The PCR mixture included 1.0 µL of each primer (10 mM) [for *nif*H: PolF 5′-TGCGAYCCSAARGCBGACTC-3′ and PolR 5′-ATSGCCATCATYTCRCCGGA-3′ [70]; and for ACCd: ACCf 5′-GCCAARCGBGAVGACTGCAA-3′ and ACCr 5′-TGCATSGAYTTGCCYTC-3′ [71], 1 µL extracted nucleotide, 9.5 µL nuclease-free water, and 12.5 µL GoTaq^®^ Green Master Mix. After PCR, the products were separated by gel electrophoresis and then stained and observed. The extracted DNA was also amplified, using the universal primers 341F 5ʹ- CCTACGGGAGGCAGCAG-3ʹ and 1378R 5ʹ-TGTGCAAGGAGCAGGGAC-3ʹ to obtain the 16S rRNA region, which was then purified and sequenced. The sequences were compared with those in the DNA Data Bank of Japan (DDBJ: http://blast.ddbj.nig.ac.jp/), and the nearest neighbor was noted. The sequences were then submitted to DDBJ.

#### 4.2.5. Plant Growth under Soil Environmental Conditions

For the vial container experiments (100 mL volume, polypropylene material), 50 g autoclaved soil with and without 200 mM NaCl (final concentration) was weighed into each vial pot. The soil moisture content was maintained at 60% of maximum water-holding capacity. Liquid fertilizer was added to the soil (a formulation of 6% N, 10% *p*, and 5% K, *w*/*v*) to achieve a final concentration of 25 mg N per 100 g soil. Two potential PGPE, strains 2 and 4, were cultured in R2B medium at 25 °C for 48 h. Three ice plant seeds were sown in the soil, and then, after 2 days, 500 µL bacterial suspension was added. Plants were grown under aseptic conditions in the growth chamber under the conditions described above at 25 °C for 32 days, and then harvested. The plant fresh and dry weights were recorded. The control plants were treated with 500 µL R2B medium without bacteria and were grown under the same conditions as those in the treatments.

### 4.3. Metabolome Analysis of PGPE Inoculated Ice Plant

*Plant growth and sample preparation:* Plants grown with two potential PGPE, strains 2 and 4, along with a mock-inoculated control, were harvested for metabolome analysis. Seeds were sown on Murashige and Skoog (MS) medium with and without NaCl (final concentration: 200 mM) and grown for 11 days under the growth conditions described in the previous section. Plant samples, including the leaves, stem, and root comprising 51, 66, 79, 58, 52, and 52 plants from the control, No. 4, No. 2, control_200 mM, No. 4_200 mM, and No. 2_200 mM treatments, respectively, were washed with sterilized distilled water, flash frozen in liquid nitrogen, and then dried in a freeze drier (FDU-12AS, AS One Corporation, Osaka, Japan). The freeze-dried samples were crushed using a Micro Smash homogenizer (TOMY SEIKO Co. Ltd., Tokyo, Japan) and weighed (9.0 mg for control plants, 11.4 mg for strain 4–treated plants, 11.9 mg for strain 2–treated plants, 10.3 mg for control plants under saline conditions, 11.5 mg for strain 4–treated plants under saline conditions, and 11.4 mg for strain 2–treated plants under saline conditions). Subsequently, each sample was homogenized (1500 rpm, 120 s) in 600 μL methanol containing internal standards (50 μM). Then, chloroform (600 μL) and Milli-Q water (240 μL) were added to the homogenate, mixed thoroughly, and then centrifuged (2300× *g*, 4 °C, 5 min). The aqueous layer (200 µL) was filtered through a 5-kDa cut-off filter (ULTRAFREE-MC-PLHCC, HMT, Millipore, Billerica, MA, USA) to remove macromolecules. The filtrate was centrifugally concentrated and resuspended in 50 μL ultrapure water immediately before analysis using capillary electrophoresis time-of-flight mass spectrometry (CE-TOFMS) in two modes to detect cationic and anionic metabolites. The analyses were conducted at Human Metabolome Technologies, Inc. (HMT) (Yamagata, Japan). The measurement conditions for the cation and anion modes of CE-TOFMS-based metabolome analysis are provided in Table S2.

*Data processing:* The peaks detected in the CE-TOFMS analysis were extracted and analyzed using automatic integration software (MasterHands 2.17.1.11, developed at Keio University) to obtain peak information, including *m*/*z*, migration time (MT), and peak area. The peak area was then converted to relative peak area using Equation (1). The peak detection limit was determined based on a signal–noise ratio of 3.

Equation (1) is as follows:

(1) $$Relative\;Peak\;Area=\frac{Metabolite\;Peak\;Area}{Internal\;Standard\;Peak\;Area\times Sample\;Amount}$$

*Annotation of peaks:* Putative metabolites were assigned by comparison with HMT’s standard library and the known–unknown peak library based on *m*/*z* and MT. The tolerance was ±0.5 min for MT and ±10 ppm (Equation (2)) for *m*/*z*. If several peaks were assigned the same candidate, the candidate was assigned separate branch numbers.

Equation (2) is as follows:

(2) $$Mass\;error\;\left(ppm\right)=\frac{Measured\;Value-Theoretical\;Value}{Measured\;Value}\times 10^{6}$$

*Quantitative estimation of target metabolites:* Absolute quantification was performed for the target metabolites. For all metabolites, the peak area was normalized to that of the internal standard, and then the concentration was calculated, using standard curves, which were obtained by single-point (100 μM) calibrations.

*Plotting on pathway map:* The putative metabolites were represented on metabolic pathway maps using VANTED (Visualization and Analysis of Networks containing Experimental Data) [72]. The pathway map was prepared based on the metabolic pathways that are known to exist, according to information in the KEGG database (http://www.genome.jp/kegg/).

### 4.4. Data Analysis

The means of PGPE effect were compared using one-way ANOVA followed by Dunnett’s test (*p* < 0.05) after confirming the normal distribution and homoscedasticity. Hierarchical cluster analysis (HCA) and principal component analysis (PCA) were performed, using statistical analysis software developed at HMT [73].

## 5. Conclusions

Our results demonstrate the plant growth-promoting effect of two bacterial endophytes (*Streptomyces* spp. strain 2 and *Microbacterium* spp. strain 4) isolated from the common ice plant at the vegetative stage. Only strain 4 showed IAA production, while strain 2 promoted plant growth but did not show IAA production or any of the other tested traits. Metabolic profiling highlighted the differences in metabolomes under salt stress and in response to inoculation with PGPE. In particular, the abundance of certain sets of metabolites, such as those related to the TCA cycle, ABC transporters, and photosynthesis, was increased in plants grown with PGPE. Salinity also affected the metabolism of ice plants, leading to changes in the contents of compatible solutes and polyamines. In contrast, many substances that were abundant in mock-inoculated salt-stressed plants showed decreased contents in salt-stressed plants inoculated with strain 2 or 4, indicating that these strains alleviated the effects of salinity stress. While the latest knowledge is accumulating [74], a deeper understanding of the complex interplay among plant metabolites may be useful for developing microbe-assisted phytoremediation strategies for saline soil.

## Figures and Tables

**Table 1:** Soil chemical properties.

		Kofu	Atagoyama	University Farmland
pH	(H_2_O)	5.6	7.6	6.6
EC	mS cm^−1^	0.16	0.31	0.07
Exchangeable CaO	mg kg^−1^	4870	13,310	2900
Exchangeable MgO	mg kg^−1^	1290	483	531
Exchangeable K_2_O	mg kg^−1^	477	517	448
CEC	cmol(+) kg^−1^	34.1	39.7	15.2
Troug-*p*	mg kg^−1^	55.7	<3.0	190
NH_4_-N	mg kg^−1^	21.4	5.2	<0.1
NO_3_-N	mg kg^−1^	18.8	41.8	25.6

## Data Availability

Not applicable.

## Supplementary Materials

The following are available online at https://www.mdpi.com/article/10.3390/ijms222111813/s1, Table S1: Heatmap and tabular presentation of all metabolites based on relative peak area in the control and strains 4- and 2-applied *Mesembryanthemum crystallinum* plants. Green (low) to red (high) represents the increase in relative area of each peak among the three treatments. Table S2: Conditions of cation and anion modes for CE-TOFMS-based metabolome analysis. Figure S1: Growth of ice plants in different soils under a range of salinity conditions. Values are means ± SD, (*n* = 3), Tukey’s HSD (*p* < 0.05). Figure S2: Initial Ice plant growth with selected strains (a), and plate photo of selected strains in this study (b).

## Abbreviations

PGPE: plant growth-promoting endophytic bacteria, IAA: indole-3-acetic acid, R2A: Reasoner’s 2A agar, PDB: potato dextrose broth, ACCd: 1-aminocyclopropane-1-carboxylate-deaminase, CE-TOFMS: Capillary Electrophoresis Time-of-Flight Mass Spectrometry, TCA cycle: tricarboxylic acid cycle, ATP: adenosine triphosphate, ADP: adenosine diphosphate, AMP: adenosine monophosphate, NADP+: nicotinamide adenine dinucleotide phosphate, UDP: uridine diphosphate, CEC: cation exchange capacity, Troug-P: Troug extractable phosphate, NH_4_-N: ammonia nitrogen, NO_3_-N: Nitrate
