# Supplementary material for: Metabolomics Analyses Reveal Metabolites Affected by Plant Growth-Promoting Endophytic Bacteria in Roots of the Halophyte Mesembryanthemum crystallinum
Source: Int J Mol Sci. 2021 Oct 30;22(21):11813. doi: 10.3390/ijms222111813 (PMC8584320; doi:10.3390/ijms222111813)

Supplementary Materials

Metabolomics analyses reveal metabolites affected by plant growth-promoting endophytic bacteria in roots of the halophyte *Mesembryanthemum crystallinum*

Ryota Kataoka[1*], Mami Akashi[1], Takeshi Taniguchi[2], Yoshiyuki Kinose[1], Ahmet Emre Yaprak[3], Oguz Can Turgay[4]

1. University of Yamanashi, Faculty of Life & Environmental Sciences, Department of Environmental Sciences, Takeda, Kofu, Yamanashi, Japan.

2. Aridland Research Center, Tottori University, Hamasaka, Tottori, Japan.

3. Ankara University, Faculty of Agriculture Department of Soil Science and Plant Nutrition, Ankara, Turkey.

4. Ankara University, Faculty of Science, Department of Biology, Ankara, Turkey.

*Corresponding Author: rkataoka@yamanashi.ac.jp, 0081-55-220-8839

Table S1 Heatmap and tabular presentation of all metabolites based on relative peak area in the control and strains 4- and 2-applied Mesembryanthemum crystallinum plants. Green (low) to red (high) represents the increase in relative area of each peak among the three treatments.

Line	ID	HMT DB			m/z	MT/RT	Standardized Relative Area					
		Compound name	PubChem CID	HMDB ID			C	4	2	C200	4200	2200
[illegible]	[illegible]	[illegible]	[illegible]	[illegible]	[illegible]	[illegible]	[illegible]	[illegible]	[illegible]	[illegible]	[illegible]	[illegible]

Table S2 Conditions of cation and anion modes for CE-TOFMS-based metabolome analysis.

**Cationic Metabolites (Cation Mode)**	
Device	Agilent CE-TOFMS system (Agilent Technologies Inc.) Machine No. 6
	Capillary: Fused silica capillary i.d. 50 μm × 80 cm
Analytical Condition	Run buffer: Cation Buffer Solution (p/n : H3301-1001)
	Rinse buffer: Cation Buffer Solution (p/n : H3301-1001)
	Sample injection: Pressure injection 50 mbar, 10 sec
	CE voltage: Positive, 30 kV
	MS ionization: ESI Positive
	MS capillary voltage: 4,000 V
	MS scan range: m/z 50-1,000
	Sheath liquid: HMT Sheath Liquid (p/n : H3301-1020)
**Anionic Metabolites (Anion Mode)**	
Device	Agilent CE-TOFMS system (Agilent Technologies Inc.) Machine No. 5
	Capillary: Fused silica capillary i.d. 50 μm × 80 cm
Analytical Condition	Run buffer: Anion Buffer Solution (p/n : H3302-1023)
	Rinse buffer: Anion Buffer Solution (p/n : H3302-1023)
	Sample injection: Pressure injection 50 mbar, 22 sec
	CE voltage: Positive, 30 kV
	MS ionization: ESI Negative
	MS capillary voltage: 3,500 V
	MS scan range: m/z 50-1,000
	Sheath liquid: HMT Sheath Liquid (p/n : H3301-1020)

Figure S1 Growth of ice plants in different soils under a range of salinity conditions. Values are means ± SD, (n=3), Tukey's HSD ($p < 0.05$).

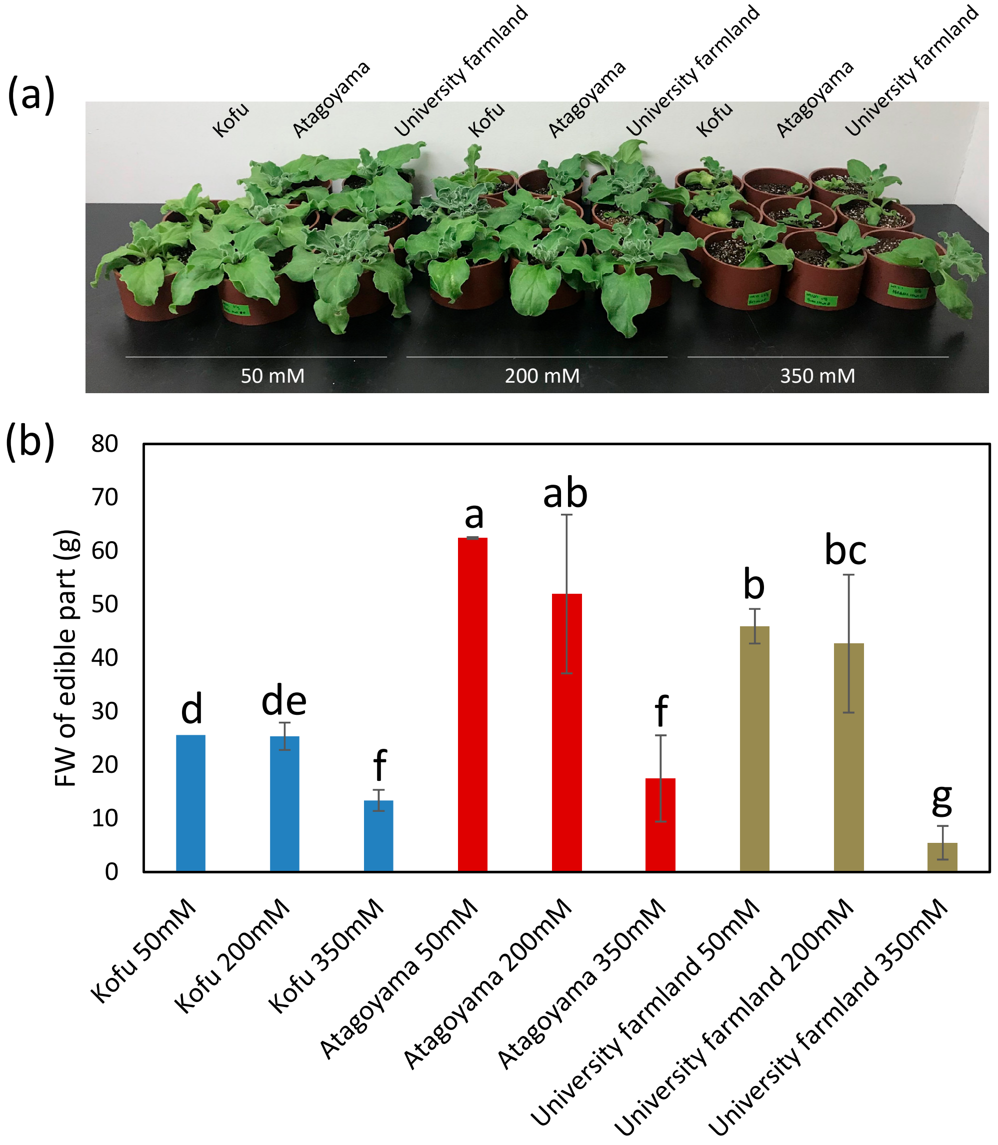

Figure S2 Initial Ice plant growth with selected strains (a), and plate photo of selected strains in this study (b).

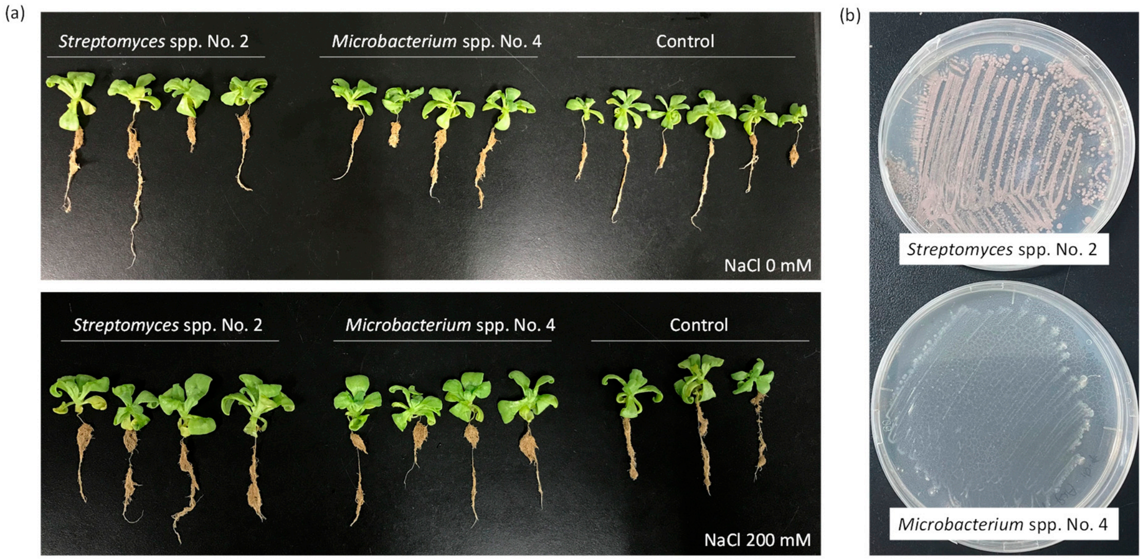

Supplement: Supplementary file 1 [file ijms-22-11813-s001.zip › ijms-1436905-supplementary.pdf]
